# Deriving Vegetation Dynamics of Natural Terrestrial Ecosystems from MODIS NDVI/EVI Data over Turkey

**DOI:** 10.3390/s8095270

**Published:** 2008-09-01

**Authors:** Fatih Evrendilek, Onder Gulbeyaz

**Affiliations:** Department of Environmental Engineering, Faculty of Engineering & Architecture, Abant Izzet Baysal University, 14280 Bolu, Turkey; E-mail: gulbeyaz_o@ibu.edu.tr (O. G.)

**Keywords:** Remote sensing, vegetation indices, ecosystem classification, spatio-temporal modeling, time series analysis

## Abstract

The 16-day composite MODIS vegetation indices (VIs) at 500-m resolution for the period between 2000 to 2007 were seasonally averaged on the basis of the estimated distribution of 16 potential natural terrestrial ecosystems (NTEs) across Turkey. Graphical and statistical analyses of the time-series VIs for the NTEs spatially disaggregated in terms of biogeoclimate zones and land cover types included descriptive statistics, correlations, discrete Fourier transform (DFT), time-series decomposition, and simple linear regression (SLR) models. Our spatio-temporal analyses revealed that both MODIS VIs, on average, depicted similar seasonal variations for the NTEs, with the NDVI values having higher mean and SD values. The seasonal VIs were most correlated in decreasing order for: barren/sparsely vegetated land > grassland > shrubland/woodland > forest; (sub)nival > warm temperate > alpine > cool temperate > boreal = Mediterranean; and summer > spring > autumn > winter. Most pronounced differences between the MODIS VI responses over Turkey occurred in boreal and Mediterranean climate zones and forests, and in winter (the senescence phase of the growing season). Our results showed the potential of the time-series MODIS VI datasets in the estimation and monitoring of seasonal and interannual ecosystem dynamics over Turkey that needs to be further improved and refined through systematic and extensive field measurements and validations across various biomes.

## 1. Introduction

Satellite observations of vegetation greenness have been used as a means to characterize amount, rate, direction, location, timing, drivers and consequences of changes in ecosystem structure and function at various spatio-temporal scales [1-6]. Seasonal and interannual vegetation dynamics and phenological patterns at the ecosystem level (e.g., timing and rate of green-up and senescence of vegetation classes, and amplitude and duration of growing season) constitute one of the key driving variables for modeling and monitoring of terrestrial ecosystems [7-9]. In response to natural and/or anthropogenic effects, the scientific community is increasingly interested in deriving information about and tracking changes in ecosystems from remotely sensed data, in order to better understand the natural and human-induced processes of changes in land cover (LC) and land use (LU) over time and space, their implications for biogeochemical cycles, and ways of interventions through management, planning, and policy to ensure sustainability of ecosystem goods and services [10-12]. Moderate Resolution Imaging Spectroradiometer (MODIS) as an instrument on board NASA's Terra and Aqua platforms for remote sensing of the atmosphere, oceans and land surfaces provides vegetation indices (VIs) more accurately than Advanced Very High Resolution Radiometer (AVHRR) as a highly evolved successor to AVHRR [13]. The two MODIS VIs of Normalized Difference Vegetation Index (NDVI) and Enhanced Vegetation Index (EVI) on a scale of minus one (-1) to plus one (+1) are spectral measures of the amount, relative greenness, phenological characteristics, and biological productivity of observed vegetation present on the ground, with a global coverage every one to two days at three spatial resolutions of 250 m, 500 m, and 1 km [14, 15]. NDVI and EVI are calculated using the following equations [13]:

(1) $$\text{NDVI}=\frac{\text{NIR}-R}{\text{NIR}+R},$$

where *NIR* and *R* stand for the spectral reflectance measurements acquired in the red and near-infrared regions, respectively.

(2) $$\text{EVI}=G\;\frac{\text{NIR}-R}{\text{NIR}+C_{1}R-C_{2}B+L},$$

where *NIR*, *R*, and *B* are reflectances in the near infrared, red, and blue bands respectively; *C*_1_ and *C*_2_ are aerosol resistance coefficients; *G* is a gain factor, and *L* is the canopy background adjustment that addresses nonlinear, differential NIR and red radiant transfer through a canopy. The coefficients adopted in the MODIS-EVI algorithm are *L* = 1, *C*_1_ = 6, *C*_2_ = 7.5, and *G* = 2.5 [16].

One of the most common VI-based approaches to quantify net and gross primary productions over time is through the following equation in process-based models [17-20]:

(3) $$\text{NPP}={ɛ}_{n}\times \text{FAPAR}\times \text{PAR}\;\text{or}\;\text{GPP}={ɛ}_{g}\times \text{FAPAR}\times \text{PAR}\;,$$

where

(4) FAPAR=a+b×NDVI,

where NPP and GPP refer to net and gross primary productivity, respectively; PAR is the incident photosynthetically active radiation (MJ m^-2^) for a given time period (e.g. hour, day or month); FAPAR is the fraction of PAR absorbed by vegetation canopy and is considered to be linearly related to NDVI; and ε_n_ and ε_g_ are the light use efficiencies (g C MJ^-1^ PAR) in the calculation of NPP and GPP, respectively. ε_n_ is the product of the potential light use efficiency (ε_0_) and reduction factors scaled to 0 to 1 that reflect environmentally limiting and reducing conditions. *a* and *b* are coefficients in the linear regression model.

Errors in VI time series are often caused by atmospheric and ground conditions (e.g. cloud and snow cover), and sensor problems (e.g. sensor drift, and changes in sensor view angle), thus creating irregularly low VI values or data gaps in time series. The time-series MODIS VI data products are, therefore, composited at 16-day intervals to minimize the degree of cloud cover by substituting a cloud covered pixel with a later uncontaminated pixel within a 16-day period. Unlike MODIS-NDVI, the MODIS-EVI accounts for the influences of dense vegetation covers, atmospheric aerosol scattering, and variable soil background reflectance [21]. The multi-temporal signatures of the time-series MODIS EVI and NDVI data were shown to capture essential phenological metrics of various natural land cover types (e.g., forest, grassland, and shrubland) and to respond differently to land cover types, canopy structures, and climate regimes [16, 22, 23].

Based on the 37-year mean climate data (1968 to 2004) from 269 meteorological stations, Dynamic Ecosystem Classification and Productivity (DECP) model developed by Evrendilek *et al.* [24] determined six biogeoclimate zones and five potential natural land cover types of Turkey according to the combination of the schemes by Holdridge [25], Box [26, 27], and the IGBP (International Geosphere-Biosphere Programme) classification system [28]. The distribution patterns of the resultant 16 natural terrestrial ecosystems (NTEs) (land use excluded) were quantified by coupling the multiple linear regression (MLR)-based interpolations of biotemperature (BT) and mean monthly temperature of the three coldest months (MMT_coldest_) as a function of digital elevation model (DEM), latitude, longitude, distance-to-sea, and aspect, and the inverse distance weighting (IDW)-based interpolation of growing season precipitation (GSP) [24]. The objective of this study was to carry out the spatio-temporal analyses of seasonal and interannual variability of vegetation dynamics for the NTEs of Turkey for the period from 2000 to 2007, based on the 16-day MODIS VI (NDVI/EVI) data. In this study, spatial disaggregating instead of the entire country was used in the form of the 16 NTE classes with different fractional tree cover for analyses of VI time series in order to differentiate among local-to-regional vegetation dynamics and to model their spatio-temporal variations for each of the NTEs.

## 2. Data and Methodology

### 2.1. Description of Study Region

**Figure 1.**
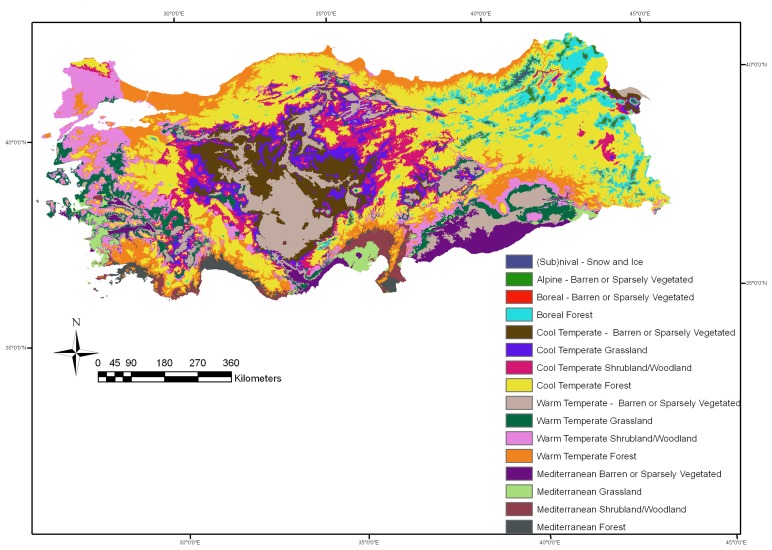
Spatial distribution pattern, location, and extent of potential natural terrestrial ecosystems [24].

Turkey (36–42°N and 26–45°E) is located where Asia, Europe, and the Middle East meet, and thus, has diverse biogeoclimatic regimes and elevation mosaic. The air temperature ranges from 45 °C in July in the southeastern region to -30 °C in February in the eastern regions. Annual precipitation varies from 258 mm in the central and southeastern regions to 2,220 mm in the northeastern Black Sea coasts. Annual evapotranspiration varies from 624 mm in the eastern region to 2,400 mm in the southeastern region. According to the long-term mean climate data between 1968 and 2004 [29], mean annual precipitation, evapotranspiration and temperature were about 634 mm, 1,280 mm, and 13 °C, respectively. The spatial distribution of the 16 NTEs is presented as determined by DECP model [24] in Figure 1. Based on the IGBP land cover classification system, barren or sparsely vegetated, grassland (steppe), woodland/shrubland and forest cover types were assumed to have the tree cover classes of <l0%, 10-30%, 30-60% and 60-100%, respectively [28].

### 2.2. Description and Preprocessing of Data

For this study, the 16-day composite MODIS NDVI and EVI products at 500-m resolution (MOD13A1 V004) were obtained for the seven-year period between February 2000 to December 2006 from the EOS Data Gateway [30]. For the coverage of Turkey, the following four tiles of the MODIS data are required: h20v04, h20v05, h21v04 and h21v05, where h and v denote the horizontal and vertical tile number, respectively. The MODIS VI datasets provided in Hierarchical Data Format (HDF) were imported to GeoTIFF format by MODIS Reprojection Tool (MRT) 3.0a [31] and reprojected from the Integerized Sinusoidal (ISIN) projection to a geographic projection (lat/lon, World Geodetic System 1984-WGS84). The MODIS VI datasets in GeoTIFF format were imported to ERDAS Imagine 8.7 [Leica Geosystems, Norcross, GA] and converted to a float data type. The entire time series of the 16-day MODIS intervals acquired for the seven-year period consisted of 161 16-day composite images (365 days yr^-1^ / 16-day composite ≅ 23 composite images yr^-1^).

The MODIS VI values equal to or below zero were assumed to be typically caused by water bodies, and thus, excluded from both seasonal and interannual curve fits by extracting pixels with negative VI values in ArcGIS 9.2 [32]. The 16-day MODIS VI datasets for the entire Turkey were spatially divided on the basis of the potential NTE distribution estimated by DECP model [24] in order for groups of biogeoclimatically similar pixels to be analyzed simultaneously in ArcGIS 9.2 [32]. The 16-day MODIS VI datasets were seasonally averaged for each of both the years and the 16 NTE types in ArcGIS 9.2 [32]. For seasonal interpretation of the results, the seasons were defined as days 65–144 (Spring = March 6 to May 24), days 145–240 (Summer = May 25 to August 28), days 241–336 (Autumn = August 29 to December 2), and days 337–64 (Winter = December 3 to March 5). The seasonally-averaged MODIS VI data were plotted in the following recurrent seasonal order of (1) Winter, (2) Spring, (3) Summer, and (4) Autumn. Seasonal and multi-year averaging helps to overcome local errors, create a baseline from which future changes can be assessed, identify vegetation characteristics and classify land cover types. Spatial disaggregating assisted in the separate analyses of time series to detect possible differences in vegetation dynamics (e.g. greenness amplitude and phase) among the NTE types with different fractional tree cover as well as to develop a generalized model to predict the spatio-tempral variation of observed differences for each of the 16 NTE classes. Spatial disaggregating has the advantage of detecting local-to-regional changes in vegetation dynamics that may hold important information about ecosystem structure and function.

### 2.3. Statistical Data Analyses

Descriptive statistics of spatially-disaggregated and temporally-aggregated time series MODIS VI data were estimated using ArcGIS 9.2 [32] as variability measures to distinguish between an unusual event and an event within the normal range of variability. Smoothing, and moving averages of the MODIS VI data were not used since VI data with sharp peaks or broad plateaus herald cases of such human-induced and/or natural disturbances as alteration of land cover and land use, defoliation, diseases, and herbivory, in their use for real-time or forecast applications. Correlations of the MODIS NDVI and EVI data were explored using Minitab 15.1 [Minitab Inc., State College, PA] according to the four land covers, six biogeoclimate zones, four seasons, and seven years.

As for the removal of periodic noise patterns in the seasonal VI time series by filtering, a discrete Fourier transformation (DFT) was adopted to decompose complex waveform domain into frequency domain. The seasonal VIs were thus separated into the signal and noise spectrums, based on the application developed by Evans and Geerken [36] with the selection of the optimal weights according to Gaussian distribution [33-36]. Fourier filtering enabled the provision of continuous time series data to estimate missing values as well as the impact of noise on the seasonal NDVI time series to be smoothed without adversely affecting the periodicity of seasonal vegetation change and the clearness of phenological characteristics [36]. The complex NDVI time series data (*V*_(_*_t_*_)_) are written in a form of discrete Fourier series as follows:

(5) $$V_{\left(t\right)}=\frac{1}{N}\sum_{x=0}^{N-1}\;f_{\left(x\right)}\cdot \exp(-i\frac{2\pi }{N}xt),$$

where *N* is the number of samples in the time series; *x* is an index representing the sample number; *f*_(_*_x_*_)_ is the *x*th sample value; *t* is the time variance in the discrete unit of season; and *i* is imaginary unit.

Comparisons between the raw and Fourier-filtered (FF) NDVI data for each NTE were made using simple linear regression (SLR) models in Minitab 15.1 [Minitab Inc., State College, PA]. The SLR models of the FF VI can be used to estimate inflection and maximum points in the FF VI time series and to delimit growing seasons. VI time series-based methods, such as sum of positive VI values over a given period, maximum value of VI over a year, (Maximum VI value − Minimum VI value) / integrated VI, slope between two VI values at two defined dates, slopes of logistic curves fitted to VI time series, threshold models, moving average procedures, number of days where NDVI > 0, number of days between the estimated date of green-up and end of the growing season, and date when the maximum VI value occurs within a year, have been commonly used to quantify annual production rate and amount of vegetation biomass, rate of spring or fall phases, start of green-up, and timing of the maximum availability of vegetation [44].

Spatial signal-to-noise ratio (SNR) variations in the MODIS VI time series were quantified to reveal efficiency of VI data in discrimination of subtle spectral responses, as follows [35, 36]:

(6) $$\text{SNR}=\frac{\text{mean}\;\text{V}{\text{I}}_{\text{signal}}}{\text{S}{\text{D}}_{\text{noise}}},$$

where SD_noise_ is the standard deviation of noise spectrum separated by Fourier filtering.

Decomposition of the MODIS VI time series with a seasonal length of four for each NTE was performed to separate the time series into the components of their linear trends and seasonally additive or multiplicative models as well as to examine the nature of the component parts in Minitab 15.1 [Minitab Inc., State College, PA]. Multi-year trends calculated for the seasonal VI time series of the 16 spatially-disaggregated ecosystem classes serve as an indicator of the direction and rate of mean seasonal change in the VI values, after the removal of seasonal effects. Similarly, seasonal indices reflect the difference of average responses for particular seasons from the overall average, after the removal of trend effects. The following three measures of accuracy of the fitted seasonal models: (1) mean absolute percentage error (MAPE); (2) mean absolute deviation (MAD); and (3) mean squared deviation (MSD), were estimated as follows:

(7) $$\begin{array}{cc} \text{MAPE}=\frac{\sum \;|\;(y_{t}-{\hat{y}}_{t})\;/y_{t}\;|}{n}x100 & \left(y_{t}\neq 0\right) \end{array},$$

(8) $$\text{MAD}=\frac{\sum_{t=1}^{n}\;\left|\;(y_{t}-{\hat{y}}_{t})\;\right|}{n},$$

(9) $$\text{MSD}=\frac{\sum_{t=1}^{n}\;{\left|\;(y_{t}-{\hat{y}}_{t})\;\right|}^{2}}{n},$$

where *y_t_* and *ŷ_t_* refer to the actual and fitted values, respectively; and *n* is the number of observations. MAPE and MAD express the accuracy of fitted time series values as a percentage and in the same units as the data, respectively. MSD is a more sensitive measure of an unusually large forecast error than MAD. For all three measures, the smaller the value is, the better the fit of the model is. MSD values are computed using the same denominator (*n*) regardless of the model, and thus, can be compared across the models.

## 3. Results and Discussion

**Figure 2.**
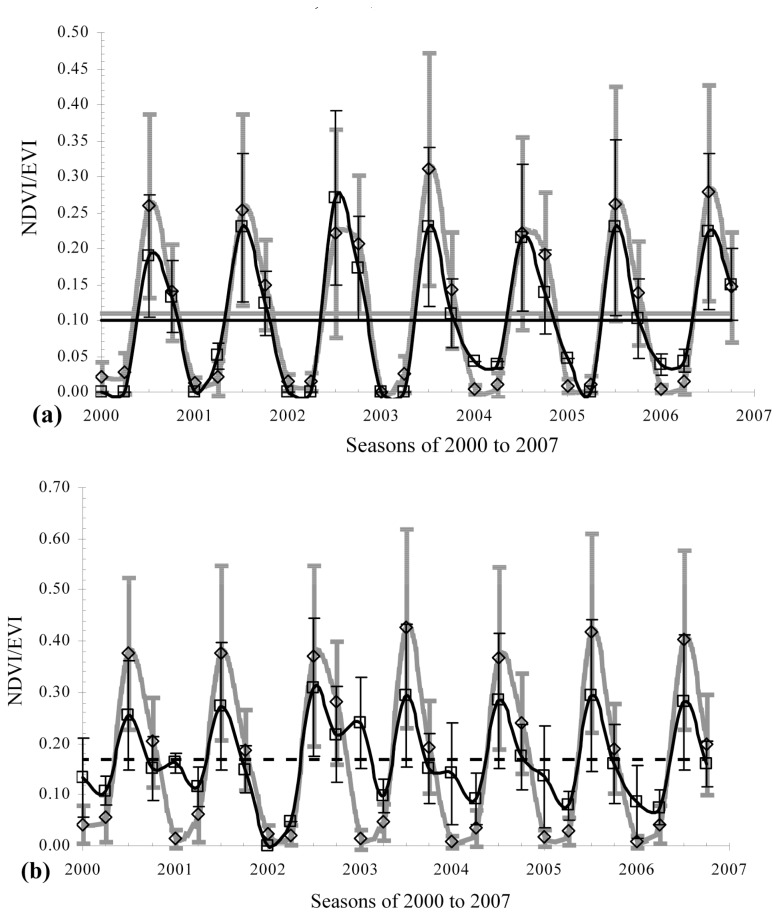

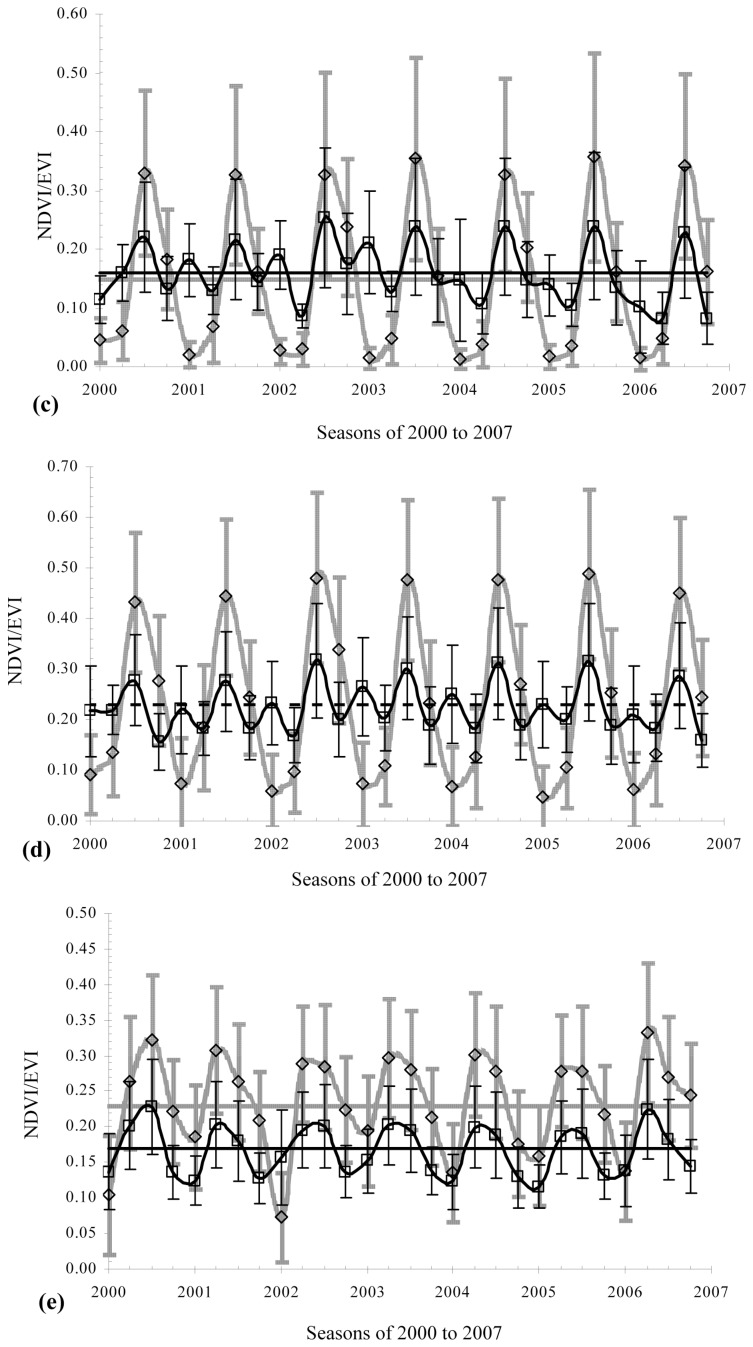

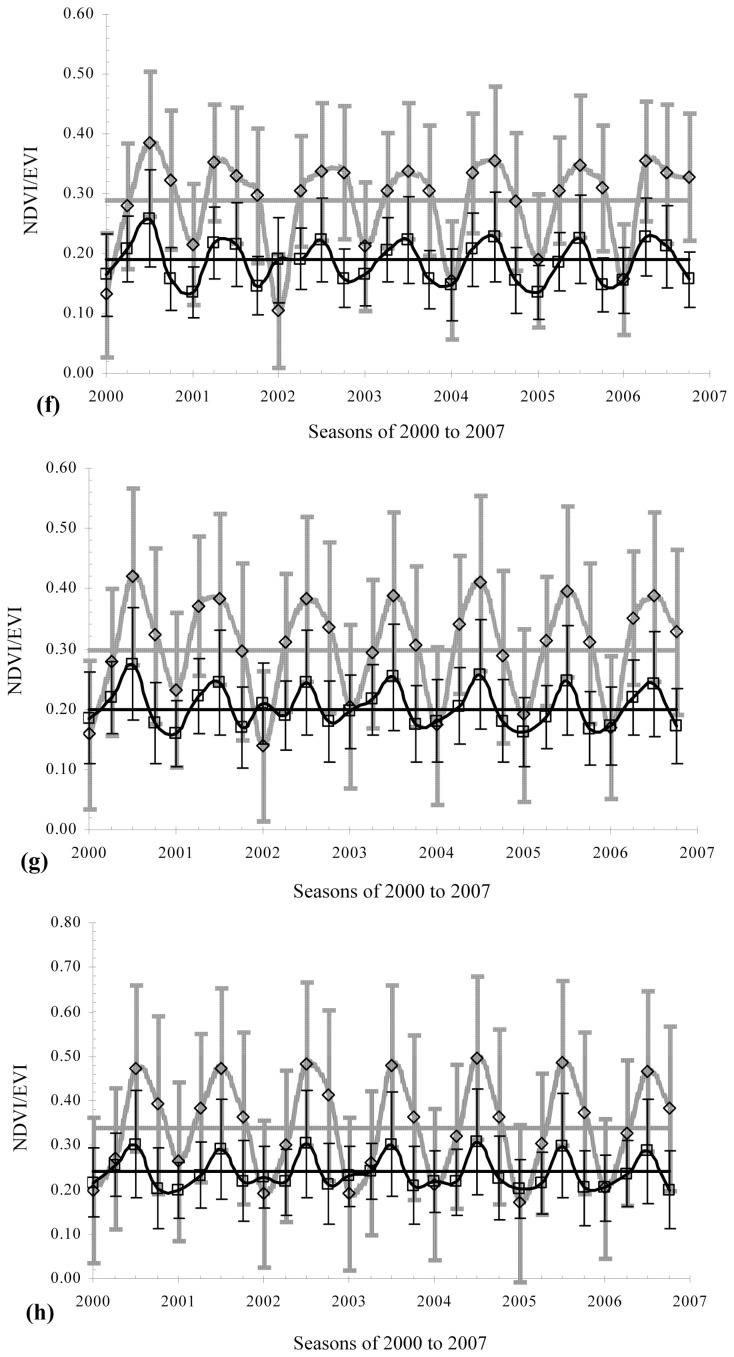

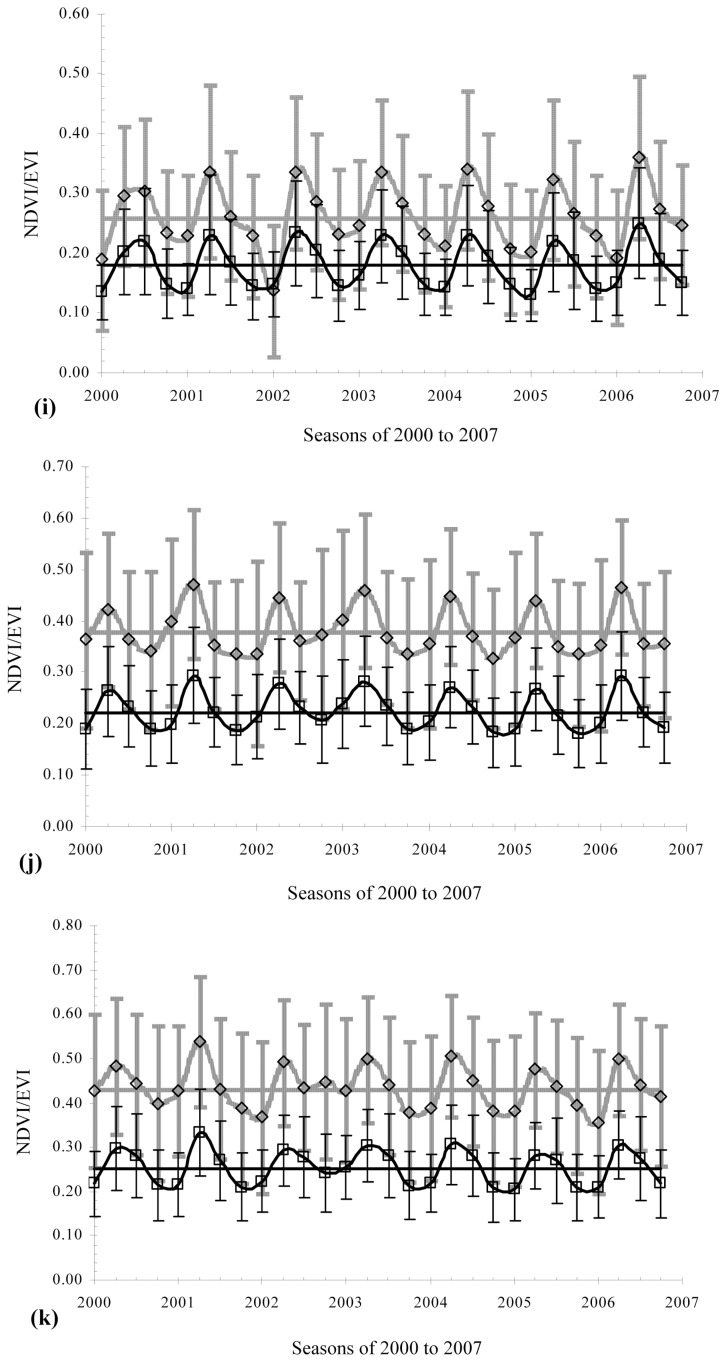

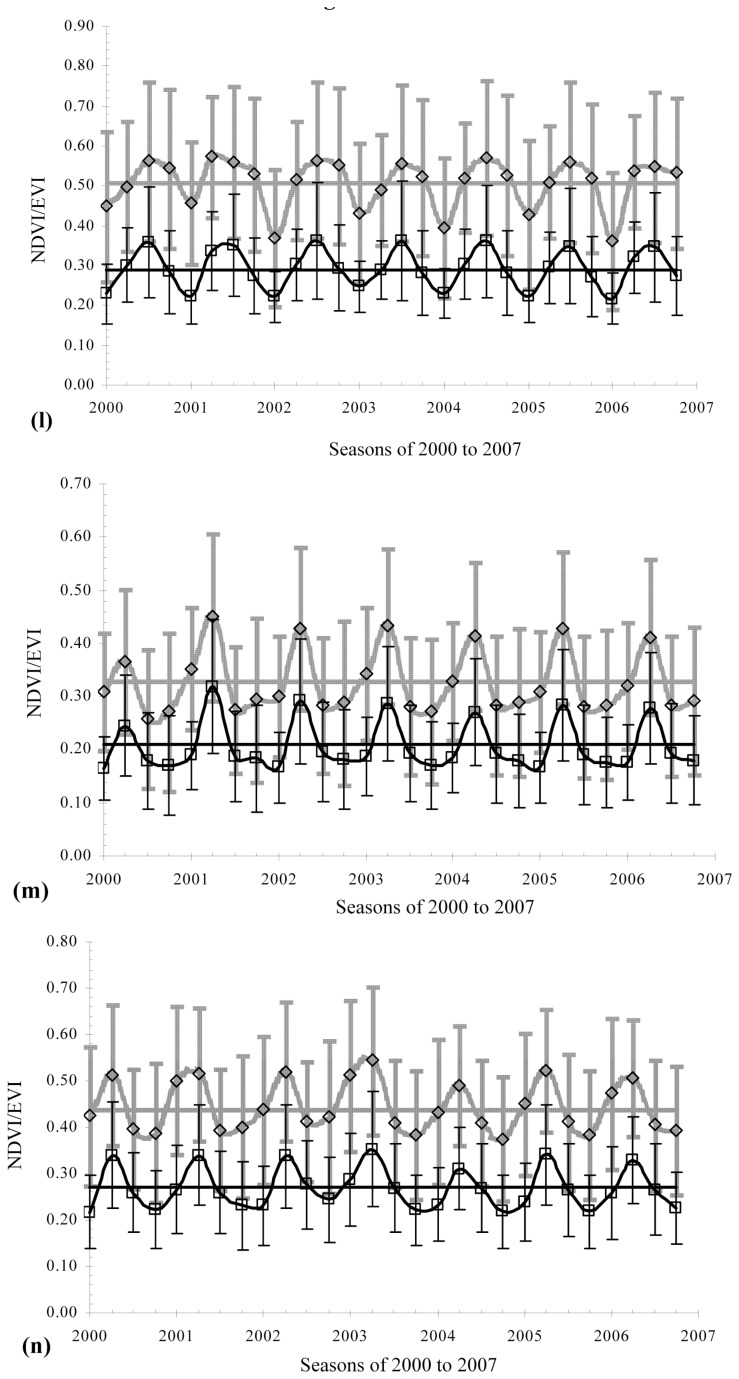

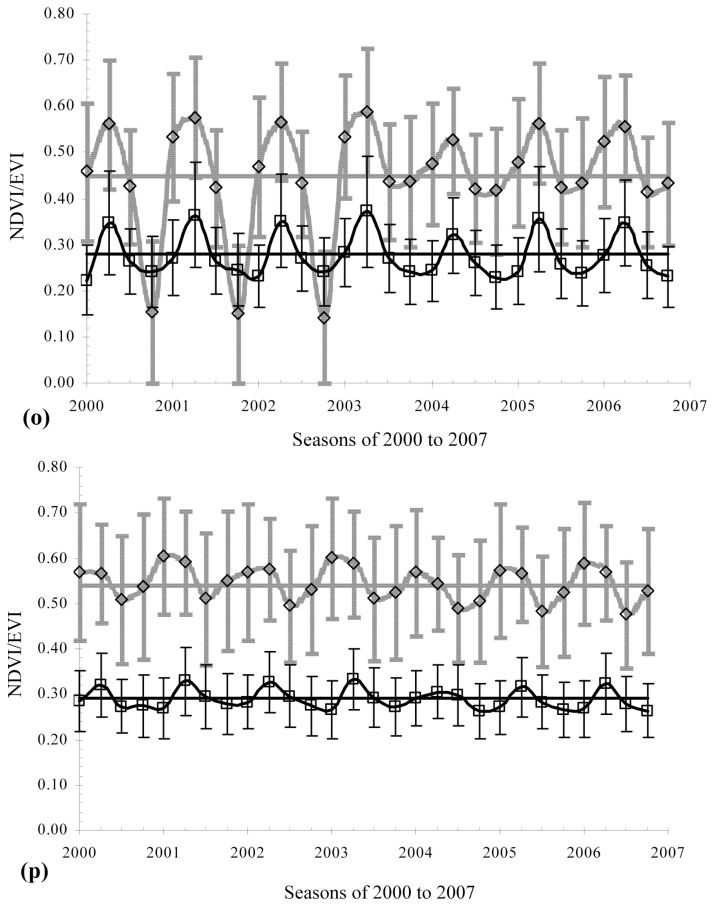
Time series MODIS NDVI (patterned curve) and EVI (solid curve) data of 2000 to 2007 for natural terrestrial ecosystems in Turkey. The recurrent seasonal order between years on X-axis is Winter, Spring, Summer, and Autumn, respectively; The error bars indicate ± one standard deviation (SD) around each mean (patterned and solid lines for NDVI and EVI, respectively, or dashed line when mean NDVI and EVI are the same); (a) (Sub)nival snow/ice; (b) Alpine barren/sparsely vegetated; (c) Boreal barren/sparsely vegetated; (d) Boreal forest; (e) Cool Temperate barren/sparsely vegetated; (f) Cool Temperate grassland; (g) Cool Temperate shrubland/woodland; (h) Cool Temperate forest; (i) Warm Temperate barren/sparsely vegetated; (j) Warm Temperate grassland; (k) Warm Temperate shrubland/woodland; (l) Warm Temperate forest; (m) Mediterranean barren/sparsely vegetated; (n) Mediterranean grassland; (o) Mediterranean shrubland/woodland; and (p) Mediterranean forest.

On average, the mean NDVI was found to maintain higher values than the mean EVI throughout the entire period for all the NTE types except for alpine and boreal barren/sparsely vegetated lands, and boreal forest (Figure 2). This general difference between the VIs found in this study supports similar findings of the related literature [16, 21-23]. Relative to the MODIS NDVI, the MODIS EVI is reported to show improved sensitivity in high biomass regions as well as an improved vegetation monitoring capability through a reduction in atmospheric impacts on the canopy background signal. The temporally averaged values of the VI time series for each NTE as shown in Table 1 can be used as a proxy to identify temporal variability in timing of onset of greenness (start of growing season) and end of growing season (onset of leaf senescence), the dates at which the VI values stay above and below the long-term mean VI level, respectively. Mean MODIS VI values were the same for alpine barren/sparsely vegetated lands, and boreal forest, while the mean NDVI value was lower than the mean EVI value for boreal barren/sparsely vegetated lands (Figure 2). The standard deviation (SD) values of the seasonal NDVI were consistently higher than those of the seasonal EVI, thus indicating the relatively high variability of the seasonal NDVI data for the NTEs in Turkey (Figure 2 and Table 1). Both VIs, on average, exhibited a unimodal growing season, with peaks in spring and summer for all the NTEs except for alpine and boreal barren/sparsely vegetated lands, and boreal and Mediterranean forest. The EVI showed a bimodal growing season, with peaks in both winter and summer for alpine and boreal barren/sparsely vegetated lands, and boreal forest, while the NDVI showed a peak in winter for Mediterranean forest only (Figure 2). Being consistent with our findings, Huete et al. [16] and Wardlow et al. [37] found both MODIS VIs to have a similar multi-temporal response over a range of LC types, with the NDVI having higher values.

In terms of potential natural LC types, biogeoclimate zones, seasons, and years, the mean VIs for the seven-year period had a similar pattern of changes in that both VIs had values in the decreasing order of forest > shrubland/woodland > grassland > barren/sparsely vegetated land > snow/ice for potential natural LC; Mediterranean > warm temperate > cool temperate > boreal > alpine > (sub)nival for biogeoclimate zones; summer > spring > autumn ≥ winter; and 2001 and 2003 ≥ 2002 > 2000 and 2004 to 2006 (Table 2). In terms of interannual variability among the LC types and biogeoclimate zones, the seasonal VIs were strongly correlated in the decreasing order of barren/sparsely vegetated land > grassland > shrubland/woodland > forests; and (sub)nival > warm temperate > alpine > cool temperate > boreal = Mediterranean, respectively (Table 3).

The VIs had the following decreasing order of correlations: summer > spring > autumn > winter; barren/sparsely vegetated land > grassland > shrubland/woodland > forests; and (sub)nival > warm temperate > alpine > cool temperate > boreal = Mediterranean. The summer, spring and autumn VIs were more strongly correlated for grassland than the other LC types, while the winter VIs were more strongly correlated for shrubland/woodland than the other LC types (Table 3). In terms of the biogeoclimate regimes, the spring, autumn, summer and winter VIs were most correlated for warm temperate, alpine, cool temperate, and winter, respectively (Table 3). The most pronounced differences between the MODIS VIs were observed in Mediterranean forest during winter, boreal forest during spring and winter, and cool temperate zone during winter.

**Figure 3.**
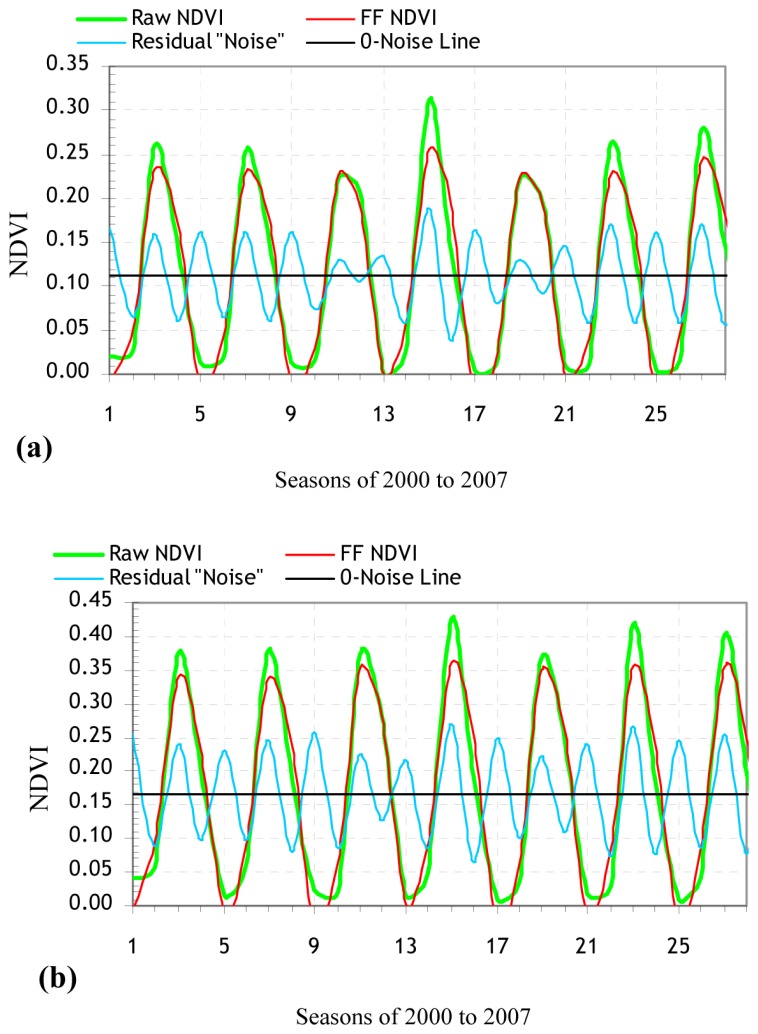

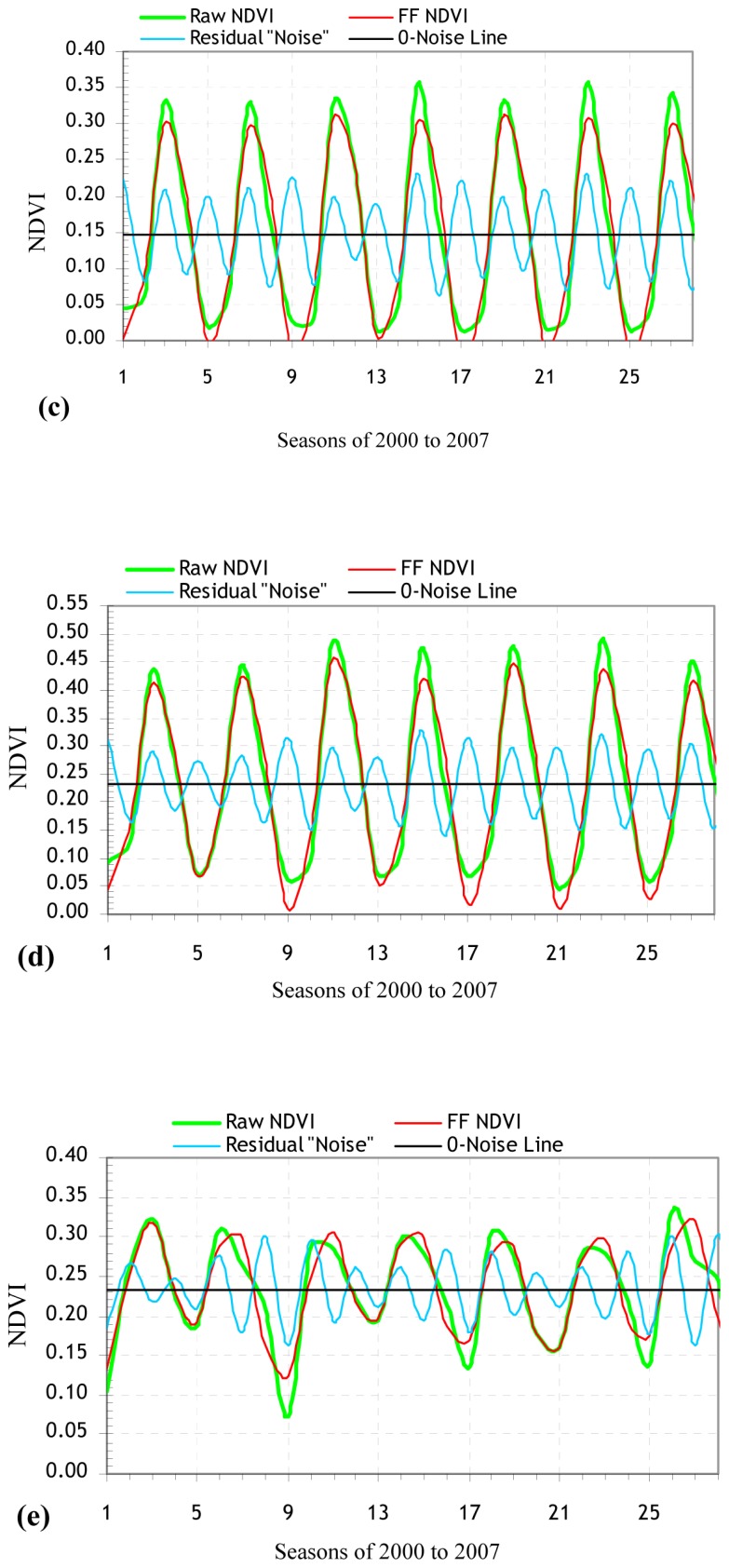

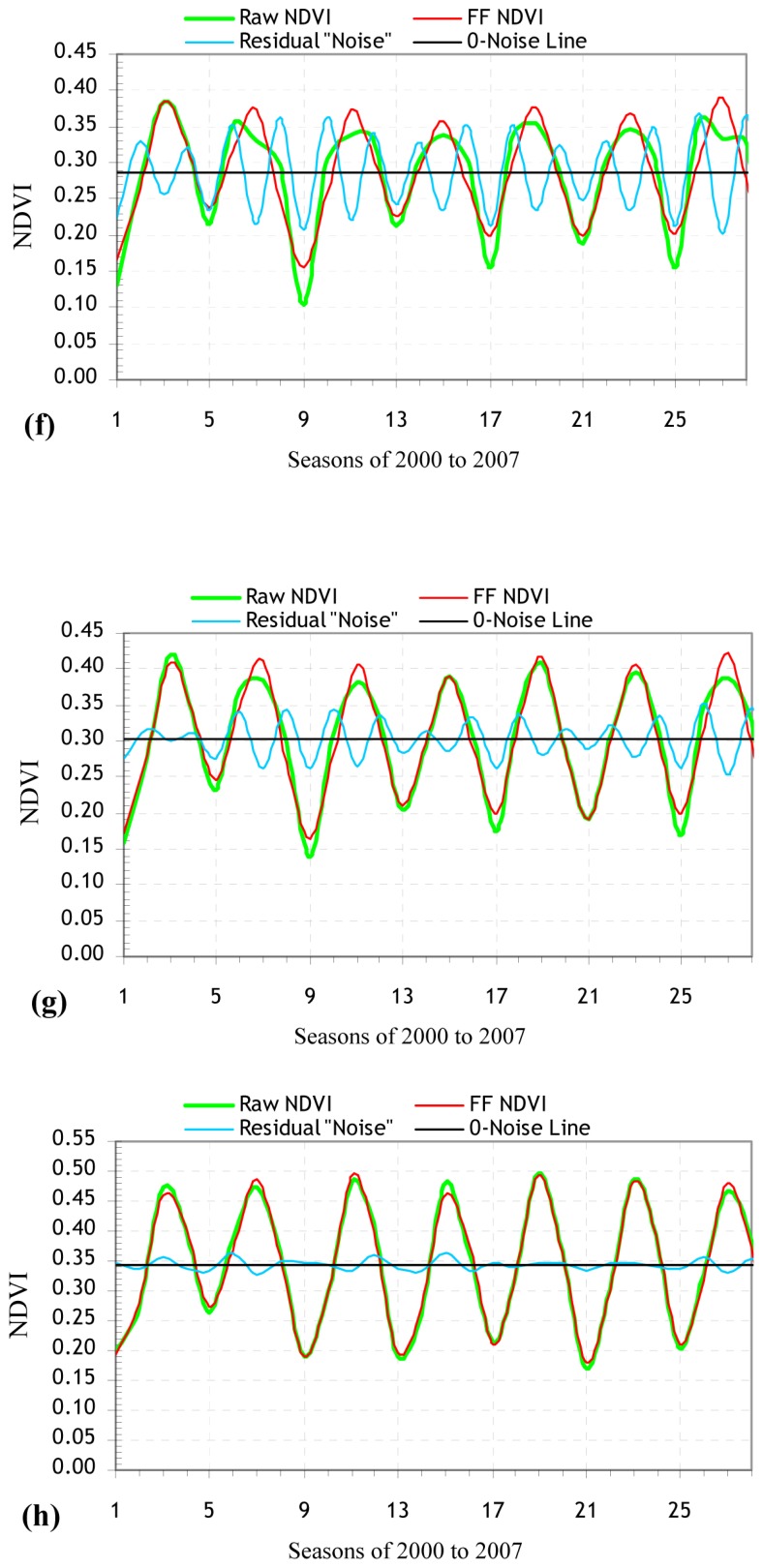

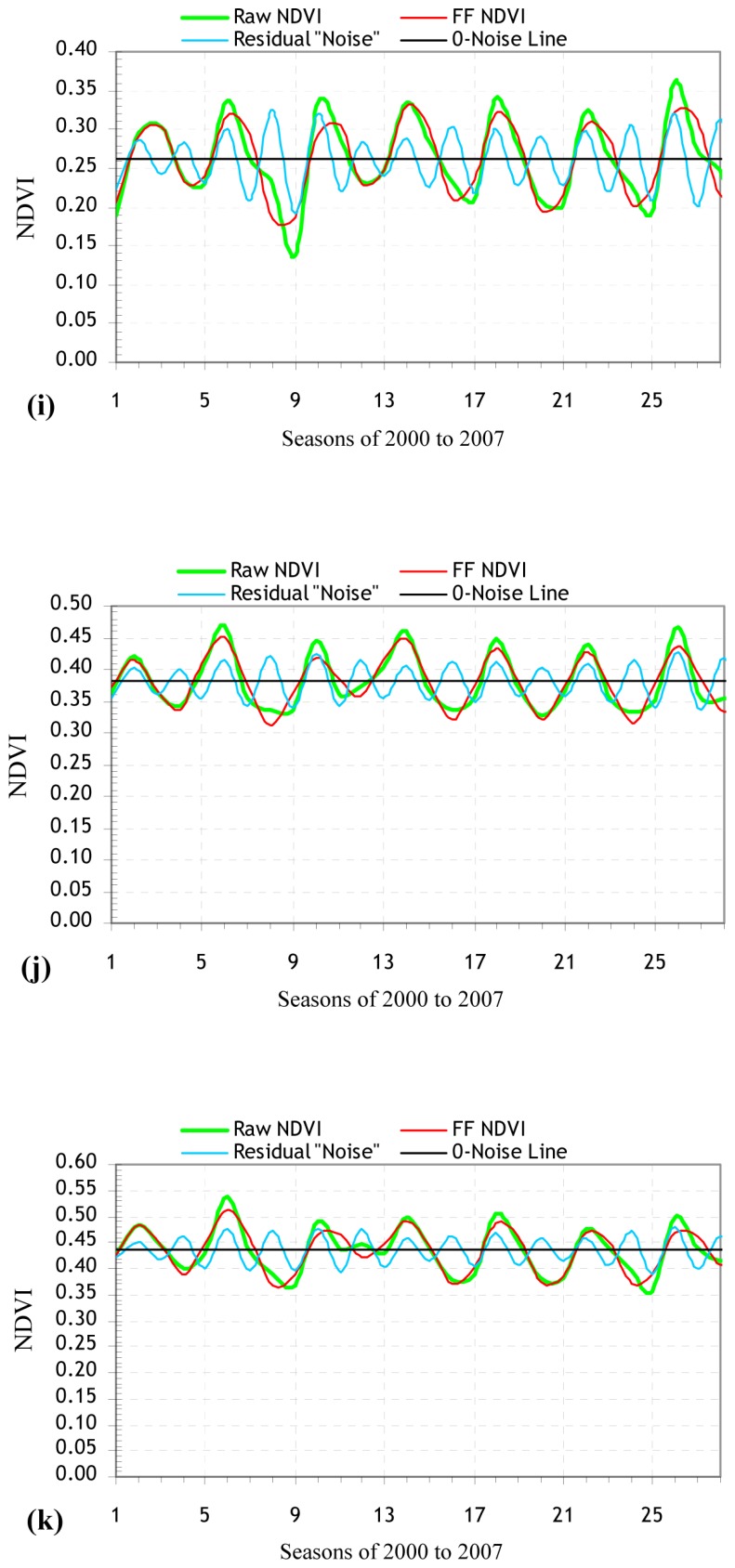

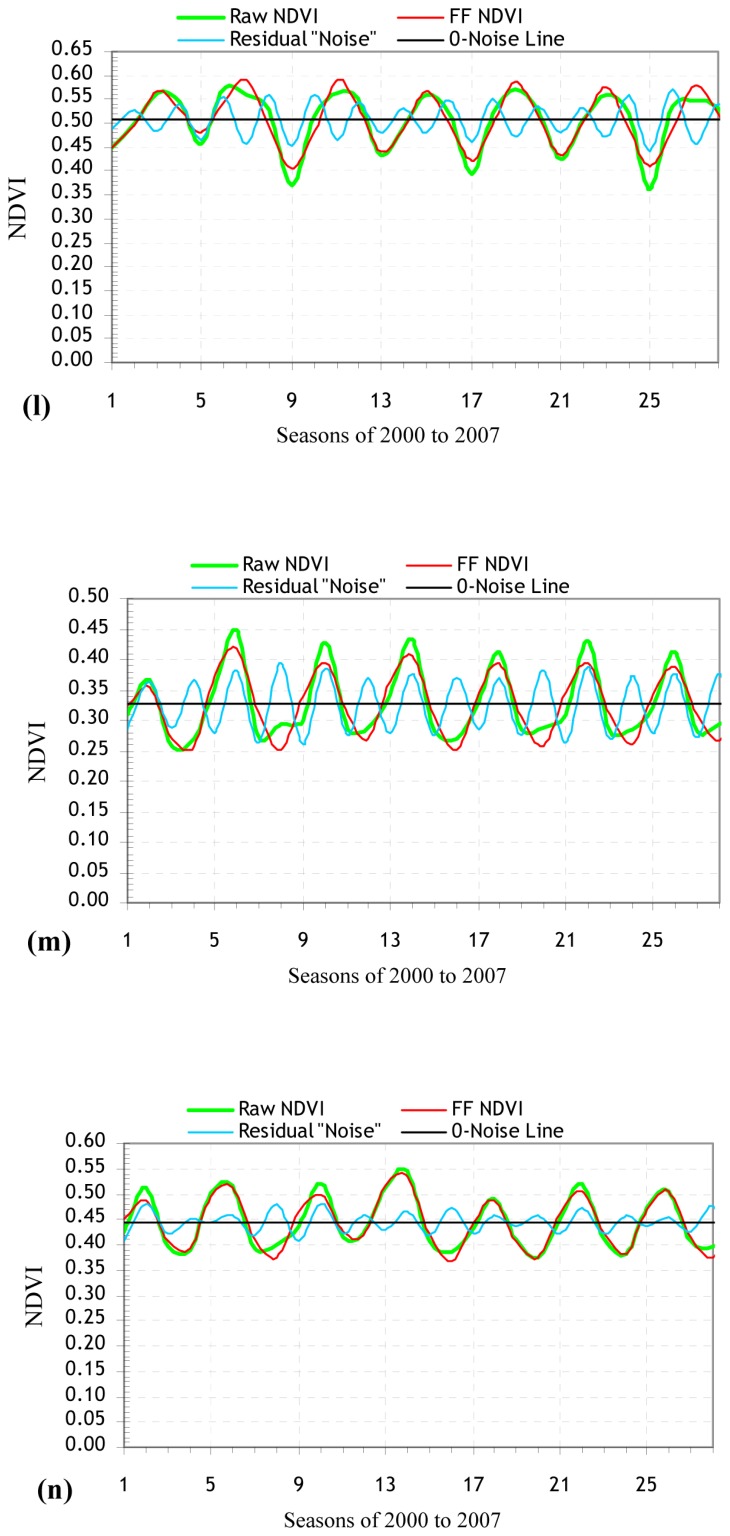

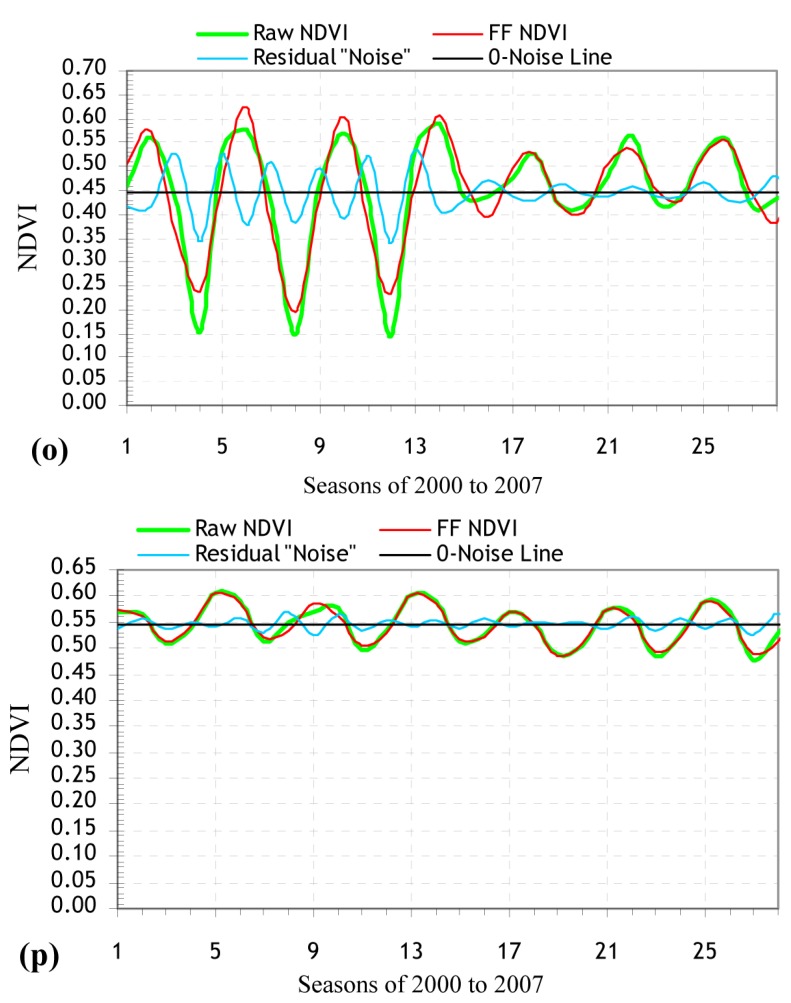
Fourier-filtered (FF) MODIS NDVI time series and their noises for natural terrestrial ecosystems in Turkey. The recurrent seasonal order between years on X-axis is Winter, Spring, Summer, and Autumn, respectively; (a) (Sub)nival snow/ice; (b) Alpine barren/sparsely vegetated; (c) Boreal barren/sparsely vegetated; (d) Boreal forest; (e) Cool Temperate barren/sparsely vegetated; (f) Cool Temperate grassland; (g) Cool Temperate shrubland/woodland; (h) Cool Temperate forest; (i) Warm Temperate barren/sparsely vegetated; (j) Warm Temperate grassland; (k) Warm Temperate shrubland/woodland; (l) Warm Temperate forest; (m) Mediterranean barren/sparsely vegetated; (n) Mediterranean grassland; (o) Mediterranean shrubland/woodland; and (p) Mediterranean forest.

**Figure 4.**
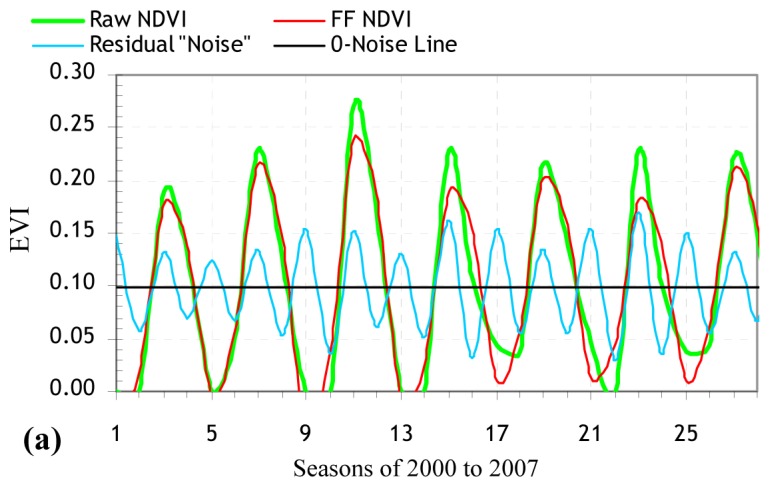

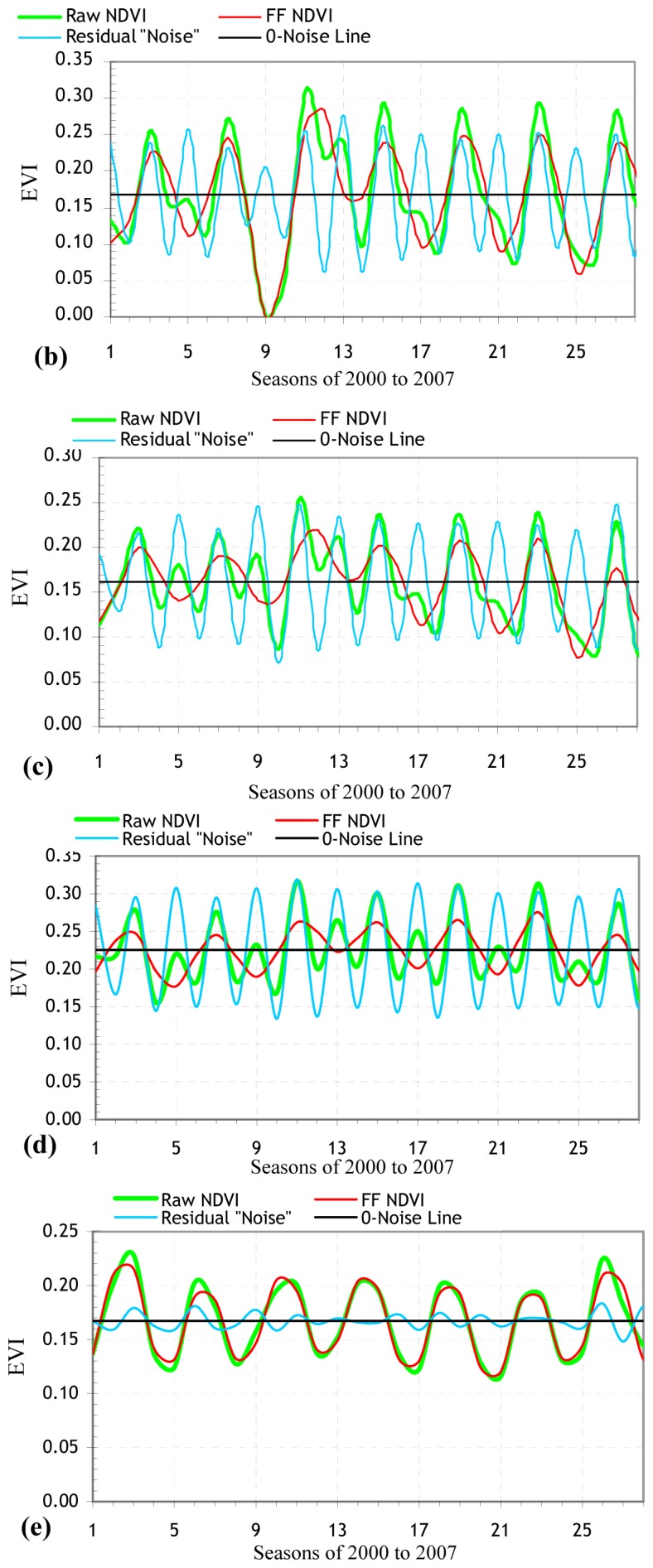

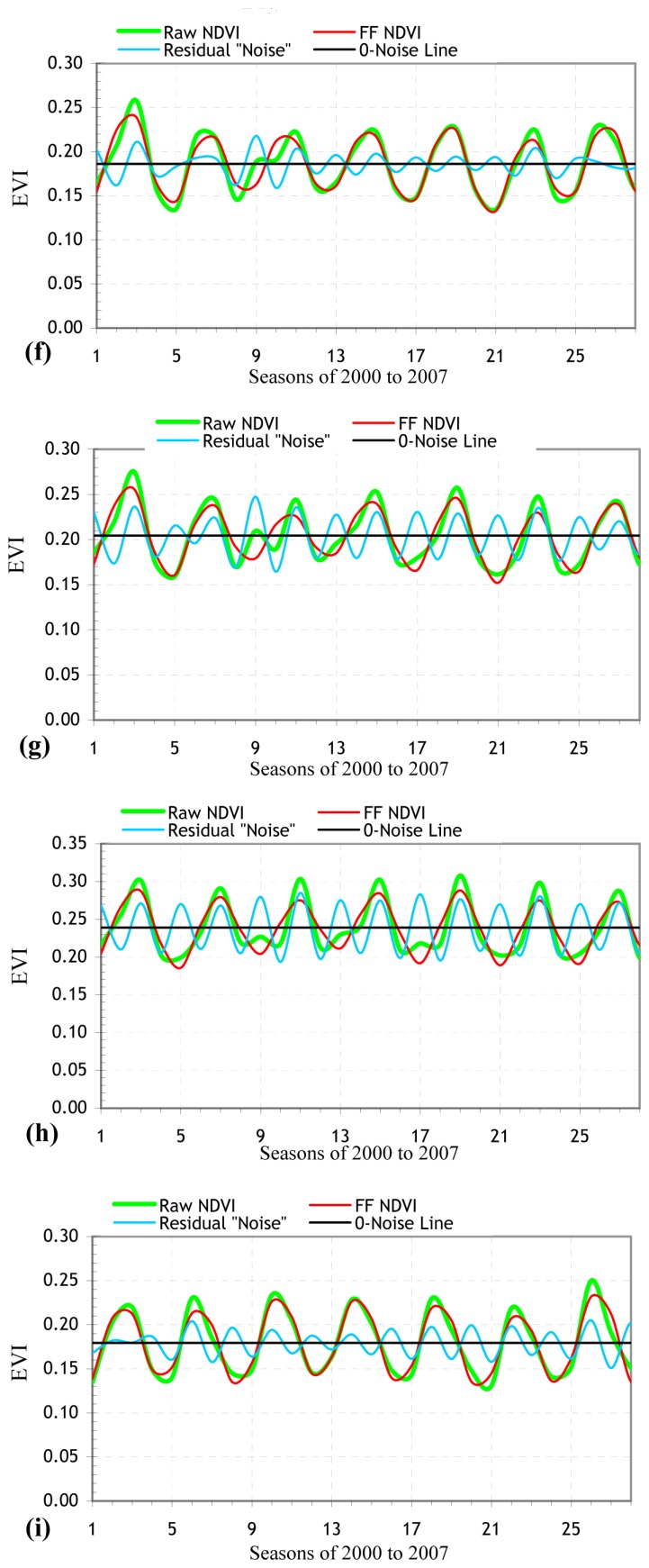

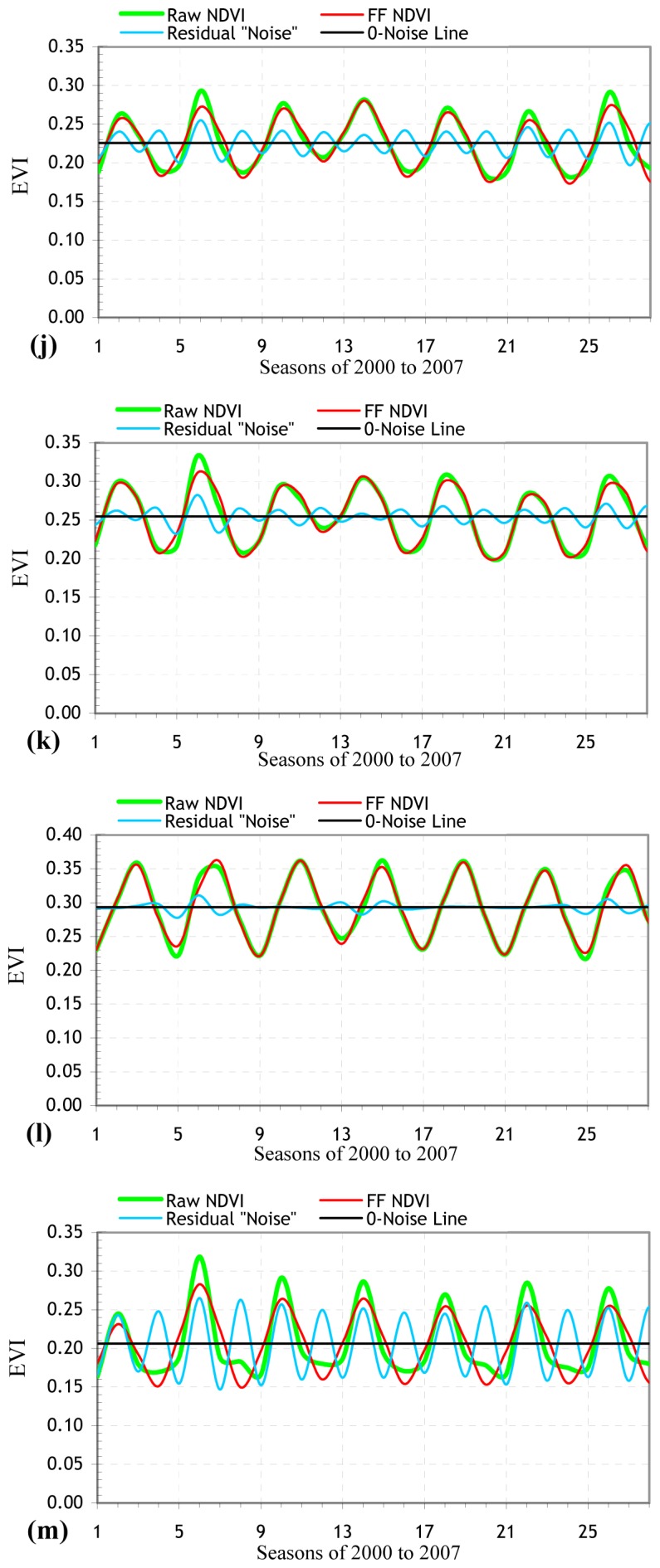

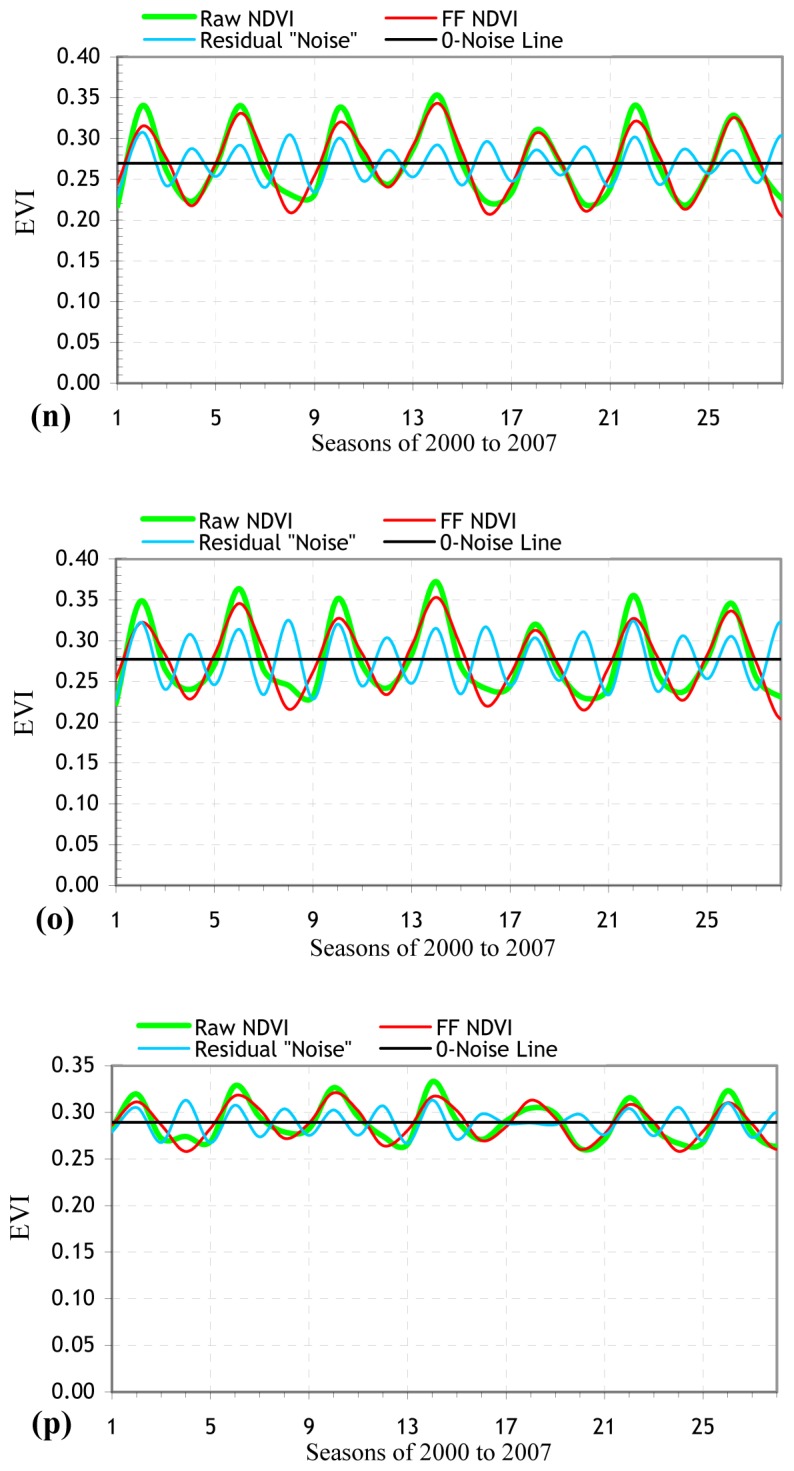
Fourier-filtered (FF) MODIS EVI time series and their noises for natural terrestrial ecosystems in Turkey.The recurrent seasonal order between years on X-axis is Winter, Spring, Summer, and Autumn, respectively; (a) (Sub)nival snow/ice; (b) Alpine barren/sparsely vegetated; (c) Boreal barren/sparsely vegetated; (d) Boreal forest; (e) Cool Temperate barren/sparsely vegetated; (f) Cool Temperate grassland; (g) Cool Temperate shrubland/woodland; (h) Cool Temperate forest; (i) Warm Temperate barren/sparsely vegetated; (j) Warm Temperate grassland; (k) Warm Temperate shrubland/woodland; (l) Warm Temperate forest; (m) Mediterranean barren/sparsely vegetated; (n) Mediterranean grassland; (o) Mediterranean shrubland/woodland; and (p) Mediterranean forest.

The DFT separated the noisy VI time series data into their individual sinusoids of different frequencies (called harmonics) and filtered the individual sinusoids to rebuild the complex waveform domain of the VI data with the periodic noise patterns of the frequencies removed (Figures 3 and 4). The remote sensing methodology is more accurate for high SNRs [35, 36]. The highest SNR values of the FF VIs associated with the NTEs were Mediterranean and cool temperate forests according to the FF NDVI, and warm temperate forest according to the FF EVI (Table 4). The SLR models for each NTE revealed the percentage of variations in the FF VI data that can be accounted for by the raw VI data, and thus, can be used to reconstruct the noise-filtered VI time series and estimate timing of start, peak, and end of as well as length of growing season (Table 4). Seasonally and annually averaged FF VI values may help to approximate the inherent cyclicity of vegetation dynamics. The seasonal SLR models of the FF VI had the highest *r*^2^ of 99.1% for cool temperate forest and 98.1% for warm temperate forest, based on the raw NDVI and EVI data (*P* < 0.001) (Table 4).

Each wave is characterized by amplitude and phase values, where the amplitude value is defined as half the height of a wave, and the phase value as range between the origin and the peak of a wave. High amplitude values for a given period show a high periodicity of, and thus, a high level of variation in the seasonal VI values. Phase values indicate the length of the time in a given year during which the VI value reaches a peak. In most VI plots where strongly periodic patterns of the seasonal VI (an indication of the presence of high amplitudes) are exhibited, there existed little or no interannual changes in annual phase values (Figures 2 to 4). The amplitudes of the seasonal NDVI data maintained higher values for all the NTE types than those of the EVI data, thus revealing a relatively wide range of the seasonal NDVI values (Figures 2 to 4).

The NTEs of (sub)nival snow/ice, and warm temperate and Mediterranean barren/sparsely vegetated lands and grasslands had similar amplitude values (Figures 2 to 4). A tendency was detected for NDVI and EVI to behave more distinctly in terms of the amplitude and phase values for alpine and boreal barren/sparsely vegetated lands, and boreal and Mediterranean forests during senescence (December to March) than the remaining NTEs during greenup (March to May). Alpine and boreal barren/sparsely vegetated lands, and boreal forest had a biomodal temporal EVI pattern that peaked in summer and winter (Figures 2 to 4). Mediterranean forest had less periodic (more chaotic) unimodal EVI and NDVI profiles that peaked in spring and winter, respectively, than the rest of the VI profiles (Figures 2 to 4).

Spatially disaggregated time series plots (Figures 2 to 4) show that the highest mean amplitudes were observed in the seasonal EVI for (sub)nival snow/ice, and alpine barren/sparsely vegetated land and in the seasonal NDVI for alpine barren/sparsely vegetated land, and boreal forest. The highest interannual variability in the amplitude value occurred for Mediterranean shrubland/woodland in the NDVI time series between the periods of 2000 to 2003 and 2003 to 2007. This transition from the high to the low NDVI amplitudes may be induced by natural events, or human management practices in Mediterranean shrubland/woodland. For example, if croplands were abandoned, or reforestation took place, then the observed NDVI variability (amplitude) might decrease as natural vegetation becomes established. The NDVI and EVI were lower than their seven-year means in both northern and high-altitude biomes (namely (sub)nival snow/ice, boreal barren/sparsely vegetated land and forest, and cool temperate barren/sparsely vegetated, grassland and shrubland/woodland). Similarly, both VIs were highest for Mediterranean and warm temperate forests and lowest for (sub)nival snow/ice, and alpine and boreal barren/sparsely vegetated lands.

Evergreen and deciduous vegetation types differ in their canopy cover and leaf longevity in that the former retains much of its canopy, thus appearing quite uniform throughout the year, whereas the latter sheds its leaves, thus changing its canopy cover widely between leaf-on and leaf-off periods. Therefore, the amplitude of the VI curves is expected to differentiate between evergreen (low amplitude) and deciduous (high amplitude) vegetation types. Both VIs showed that Mediterranean forest, where evergreen coniferous trees (e.g. *Pinus*) dominate, had the lowest amplitude throughout the entire period. Similarly, boreal forest with conifers (e.g. *Picea* and *Abies*) had a pronounced bimodal growing season with low amplitudes according to the EVI. However, in cool and warm temperate forests where the high proportion of deciduous vegetation or its pure stands are dominant, the amplitude of the NDVI and EVI increased.

Time series decomposition of the VIs revealed that the seven-year trends linearly fitted differed in the direction of change between the VIs for the six NTEs of (sub)nival ice/snow, boreal forest, cool temperate barren/sparsely vegetated land, grassland and shrubland/woodland, and Mediterranean shrubland/woodland (Tables 5 and 6). When the three accuracy measures (MAPE, MAD, and MSD) of the trend equations were compared between the VIs, the trends fitted to the NDVI time series were found to perform better for the three NTEs of (sub)nival snow/ice, and alpine and boreal barren/sparsely vegetated lands than the EVI trends (Tables 5 and 6).

The VI trends with the lowest error statistics pointed to the negative NDVI trends for the three highest-altitude NTEs of (sub)nival, alpine and boreal barren/sparsely vegetated lands; the positive EVI trends for the four NTEs of boreal forest, warm temperate and Mediterranean barren/sparsely vegetated lands, and Mediterranean grassland; and the negative EVI trends for the rest of the NTEs (Tables 5 and 6). The negative VI trends during the seven-year period can indicate increased natural and/or human-induced disturbances of vegetation cover qualitatively and quantitatively such as deforestation, and conversion of land cover from woody to non-woody vegetation [3, 38-43].

The positive VI trends can indicate increased VI levels observed over the course of growing season, an increased capacity to fix carbon via photosynthesis, increased vegetation density and canopy cover, and progressive succession and may even reflect positive side effects of climate warming in the northern and/or high-altitude NTEs [38-43]. The highest seasonal indices of both VIs occurred consistently during the summer and spring except for Mediterranean forest in the winter according to the NDVI, thus indicating the higher VI values in the summer and spring, on average, than the rest for the NTEs (Tables 5 and 6).

## 4. Conclusions

Gathering more accurate, detailed and timely information about the state of the environment where ground knowledge is sparse requires environmental monitoring through remotely sensed VIs at the spatio-temporal scales to support the demands of decision- and policy-makers towards proactive, rational and adaptive strategies. Our results indicate that national classification of the 16 potential NTE types by DECP model [24] is consistent with such vegetation metrics derived from the seasonally aggregated MODIS VIs as mean, minimum and maximum VIs, and VI amplitudes and phases. Our coupled graphical and statistical analyses of the 16-day MODIS VI profiles seasonally averaged on the basis of the NTEs reflected differences in climate zones, land cover types, seasons, years, leaf longevity (deciduous vs. evergreen vegetation), permanence of vegetation (woody vs. nonwoody vegetation), and disturbances of vegetation cover. In Turkey, both MODIS VIs, on average, depicted similar seasonal variations for the NTEs, with the NDVI values having higher mean and SD values. Differences between the MODIS VI responses were most pronounced in boreal and Mediterranean climate zones and forests, and in winter (the senescence phase of the growing season) over Turkey. These findings need to be validated through systematic and extensive field measurements across various biomes of Turkey. This would improve and refine the potential of the time-series MODIS VI datasets for the estimation of seasonal and interannual ecosystem dynamics in Turkey.

## Figures and Tables

**Table 1.** Biogeoclimate zones and natural land cover types of Turkey, and their extent, biophysical properties and MODIS-derived vegetation indices.

*Biogeoclimate zones*	*Land cover types*	*Number of pixels*	*Area (km^2^)*	*Mean NDVI*	*Mean EVI*	*Mean altitude (m)*	*BT (*°*C)*	*PER*
(Sub)nival	Snow/ice	5899	1475	0.11 ± 0.06	0.10 ± 0.04	3104 ± 258	0.6 ± 0.9	0.27 ± 0.14
Alpine	Barren/sparsely vegetated	20372	5093	0.17 ± 0.08	0.17 ± 0.07	2831 ± 220	2.3 ± 0.4	0.33 ± 0.10
Boreal	Barren/sparsely vegetated	9043	2261	0.15 ± 0.08	0.16 ± 0.07	2391 ± 264	4.8 ± 0.8	0.52 ± 0.14
	Forest	167254	41814	0.23 ± 0.11	0.23 ± 0.08			
Cool Temperate	Barren/sparsely vegetated	212169	53042	0.23 ± 0.08	0.17 ± 0.05	1423 ± 413	9.6 ± 1.5	1.11 ± 0.35
	Grassland	175572	43893	0.29 ± 0.11	0.19 ± 0.06			
	Shrubland/woodland	278098	69525	0.30 ± 0.13	0.20 ± 0.07			
	Forest	979753	244938	0.34 ± 0.18	0.24 ± 0.09			
Warm Temperate	Barren/sparsely vegetated	312485	78121	0.26 ± 0.11	0.18 ± 0.07	663 ± 371	13.5 ± 1.1	1.40 ± 0.41
	Grassland	162401	40600	0.38 ± 0.14	0.22 ± 0.08			
	Shrubland/woodland	281280	70320	0.43 ± 0.15	0.25 ± 0.08			
	Forest	322362	80591	0.51 ± 0.18	0.29 ± 0.10			
Mediterranean	Barren/sparsely vegetated	101905	25476	0.33 ± 0.13	0.21 ± 0.09	303 ± 211	17.3 ± 1.0	1.59 ± 0.45
	Grassland	55559	13890	0.44 ± 0.15	0.27 ± 0.09			
	Shrubland/woodland	54595	13649	0.45 ± 0.13	0.28 ± 0.08			
	Forest	43459	10865	0.54 ± 0.13	0.29 ± 0.06			
Grand total/mean		3182206	795552	0.32 ± 0.13	0.21 ±0.05	1141 ± 655	11.3 ± 3.3	1.21 ± 0.44

BT: Biotemperature; PER: Ratio of potential evapotranspiration to precipitation. Mean NDVI and EVI values are for the period of 2000 to 2007.

**Table 2.** MODIS NDVI and EVI properties in response to changes in natural land cover, biogeoclimate zone, season, and year.

Four categories of explanatory variables	EVI				NDVI			

	mean	± SD	min	max	mean	± SD	min	max
*Natural Land Cover*								
Snow/ice	0.10	0.04	0.02	0.23	0.15	0.08	0.00	0.45
Barren/sparsely vegetated	0.18	0.07	0.02	0.52	0.23	0.10	0.00	0.68
Grassland	0.23	0.08	0.01	0.59	0.37	0.13	0.00	0.86
Shrubland/woodland	0.24	0.08	0.01	0.60	0.40	0.14	0.00	0.87
Forest	0.26	0.08	0.01	0.61	0.41	0.15	0.00	0.87
*Biogeoclimate Zone*								
(Sub)nival	0.10	0.04	0.02	0.23	0.11	0.06	0.00	0.34
Alpine	0.17	0.07	0.03	0.42	0.17	0.08	0.01	0.48
Boreal	0.19	0.08	0.03	0.50	0.19	0.10	0.01	0.64
Cool Temperate	0.20	0.07	0.01	0.57	0.29	0.12	0.00	0.85
Warm Temperate	0.24	0.08	0.00	0.62	0.40	0.15	0.00	0.88
Mediterranean	0.26	0.08	0.01	0.60	0.44	0.14	0.00	0.87
*Season*								
Winter	0.19	0.06	0.01	0.49	0.25	0.11	0.00	0.72
Spring	0.23	0.07	0.01	0.55	0.33	0.11	0.00	0.72
Summer	0.25	0.09	0.01	0.67	0.39	0.14	0.00	0.87
Autumn	0.19	0.07	0.01	0.50	0.32	0.13	0.00	0.81
*(Inter)annual*								
2000	0.21	0.07	0.01	0.55	0.32	0.13	0.00	0.80
2001	0.22	0.07	0.01	0.56	0.33	0.12	0.00	0.79
2002	0.22	0.07	0.01	0.55	0.32	0.12	0.00	0.78
2003	0.22	0.08	0.02	0.56	0.33	0.12	0.00	0.78
2004	0.21	0.07	0.01	0.55	0.32	0.12	0.00	0.77
2005	0.21	0.07	0.01	0.54	0.32	0.12	0.00	0.77
2006	0.21	0.07	0.01	0.55	0.32	0.12	0.00	0.77
Grand mean	0.21	0.07	0.01	0.55	0.32	0.12	0.00	0.78

**Table 3.** Correlation of MODIS NDVI and EVI values according to natural land cover types, biogeoclimate zones, and seasons in Turkey.

Natural terrestrial ecosystem (NTE) types	NTE types		Winter		Spring		Summer		Autumn	
	*r*	*P*	*r*	*P*	*r*	*P*	*r*	*P*	*r*	*P*
(Sub)nival snow/ice	0.96	<0.001	-0.44	>0.05	-0.25	>0.05	-0.24	>0.05	0.74	0.05
Alpine barren/sparsely vegetated	0.85	<0.001	-0.17	>0.05	0.90	0.005	0.25	>0.05	0.95	0.001
Boreal barren/sparsely vegetated	0.72	<0.001	-0.24	>0.05	0.70	>0.05	0.20	>0.05	0.62	>0.05
Boreal forest	0.60	0.001	-0.06	>0.05	-0.02	>0.05	0.95	0.001	0.35	>0.05
Cool Temperate barren/sparsely vegetated	0.81	<0.001	-0.37	>0.05	0.74	0.05	0.98	<0.001	0.77	0.04
Cool Temperate grassland	0.58	0.001	-0.69	>0.05	0.67	>0.05	0.96	<0.001	0.52	>0.05
Cool Temperate shrubland/woodland	0.66	<0.001	-0.64	>0.05	0.20	>0.05	0.91	0.004	0.07	>0.05
Cool Temperate forest	0.69	<0.001	-0.39	>0.05	-0.30	>0.05	0.84	0.01	-0.34	>0.05
Warm Temperate barren/sparsely vegetated	0.93	<0.001	0.21	>0.05	0.98	<0.001	0.98	<0.001	0.29	>0.05
Warm Temperate grassland	0.91	<0.001	0.38	>0.05	0.94	0.001	0.89	0.006	0.94	0.001
Warm Temperate shrubland/woodland	0.92	<0.001	0.51	>0.05	0.97	<0.001	0.61	>0.05	0.93	0.002
Warm Temperate forest	0.87	<0.001	0.36	>0.05	0.96	<0.001	0.69	>0.05	0.76	0.04
Mediterranean barren/sparsely vegetated	0.92	<0.001	0.95	0.001	0.94	0.001	0.95	0.001	0.96	<0.001
Mediterranean grassland	0.85	<0.001	0.97	<0.001	0.94	0.001	0.84	0.01	0.98	<0.001
Mediterranean shrubland/woodland	0.62	<0.001	0.97	<0.001	0.99	<0.001	0.90	0.005	-0.67	>0.05
Mediterranean forest	0.26	>0.05	-0.79	0.03	0.95	0.001	0.14	>0.05	0.86	0.01
Mean ± SD	0.76 ± 0.19		0.04 ± 0.60		0.64 ± 0.46		0.68 ± 0.38		0.55 ± 0.49	

**Table 4.** Comparison of MODIS-NDVI filtered by the Discrete Fourier Transform (DFT) versus raw NDVI, and spatial variations of signal-to-noise ratio (SNR) in Fourier-filtered (FF) NDVI for natural terrestrial ecosystems in Turkey.

Natural terrestrial ecosystem types	Regression coefficients		*r*^2^ (%)	SNR	Regression coefficients		*r*^2^ (%)	SNR
	
	Intercept	Raw NDVI			Intercept	Raw EVI		
(Sub)nival snow/ice	0.0087	0.919	93.1	2.26	0.00911	0.904	90.9	2.00
Alpine barren/sparsely vegetated	0.0145	0.912	92.5	2.08	0.0469	0.719	73.2	2.03
Boreal barren/sparsely vegetated	0.0130	0.910	92.3	2.17	0.0828	0.485	52.9	2.30
Boreal forest	0.0161	0.929	94.1	3.24	0.156	0.308	32.7	2.81
Cool Temperate barren/sparsely vegetated	0.0424	0.818	82.7	4.97	0.0121	0.927	94.4	19.92
Cool Temperate grassland	0.0603	0.789	79.8	4.58	0.0231	0.875	90.0	12.37
Cool Temperate shrubland/woodland	0.0215	0.929	94.0	9.32	0.0439	0.784	80.7	7.60
Cool Temperate forest	0.0062	0.981	99.1	34.55	0.0640	0.732	75.2	6.51
Warm Temperate barren/sparsely vegetated	0.0575	0.780	79.1	6.00	0.0177	0.901	91.8	10.38
Warm Temperate grassland	0.0667	0.824	83.7	11.31	0.0248	0.890	90.9	11.75
Warm Temperate shrubland/woodland	0.0730	0.832	84.1	13.09	0.0153	0.940	95.9	19.92
Warm Temperate forest	0.0852	0.832	84.4	12.07	0.0115	0.961	98.1	41.89
Mediterranean barren/sparsely vegetated	0.0700	0.785	79.5	6.25	0.0583	0.717	72.7	4.32
Mediterranean grassland	0.0380	0.914	92.8	18.77	0.0321	0.880	89.3	10.28
Mediterranean shrubland/woodland	0.0707	0.843	86.3	8.58	0.0540	0.804	81.6	7.18
Mediterranean forest	0.0361	0.933	95.5	43.66	0.0594	0.794	81.0	17.75

FF: Fourier filtered; SNR: signal-to-noise ratio; all the linear regression models and coefficients of raw NDVI and EVI are statistically significant at *P* < 0.001.

**Table 5.** Decomposition of MODIS NDVI time series into trend and seasonal components for natural terrestrial ecosystems in Turkey.

*Natural terrestrial ecosystem types*	*Components*		*Seasonal Indices*				*Accuracy measurements*		
	Trend	Seasonal	W	Sp	Su	A	MAPE	MAD	MSD
(Sub)nival snow/ice	0.1125 - 0.00010*t	A	-0.0991	-0.0903	0.1494	0.0401	672.52	0.013	0.0001
Alpine barren/sparsely vegetated	0.1682 - 0.00012*t	A	-0.1437	-0.1203	0.2213	0.0428	29.40	0.016	0.0005
Boreal barren/sparsely vegetated	0.1533 - 0.00044*t	A	-0.1233	-0.0973	0.1871	0.0336	18.71	0.013	0.0003
Boreal forest	0.2408 - 0.00065*t	A	-0.1666	-0.1097	0.2416	0.0348	10.15	0.017	0.0006
Cool Temperate barren/sparsely vegetated	0.2269 + 0.00047*t	A	-0.0852	0.0651	0.0453	-0.0252	11.72	0.019	0.0007
Cool Temperate grassland	0.2785 + 0.00048*t	M	0.5975	1.1402	1.1892	1.0729	9.47	0.020	0.0007
Cool Temperate shrubland/woodland	0.3004 + 0.00018*t	A	-0.1230	0.0267	0.0920	0.0042	7.36	0.019	0.0005
Cool Temperate forest	0.3480 - 0.00031*t	A	-0.1412	-0.0286	0.1422	0.0276	6.31	0.018	0.0006
Warm Temperate barren/sparsely vegetated	0.2560 + 0.00033*t	M	0.7975	1.2783	1.0589	0.8652	6.58	0.014	0.0004
Warm Temperate grassland	0.3814 - 0.00007*t	A	-0.0132	0.0716	-0.0187	-0.0396	3.14	0.012	0.0002
Warm Temperate shrubland/woodland	0.4446 - 0.00071*t	A	-0.0388	0.0698	0.0077	-0.0387	3.74	0.016	0.0004
Warm Temperate forest	0.5199 - 0.00092*t	A	-0.0959	0.0148	0.0561	0.0248	3.52	0.016	0.0004
Mediterranean barren/sparsely vegetated	0.3196 + 0.00047*t	M	0.9953	1.2961	0.8493	0.8590	3.47	0.011	0.0002
Mediterranean grassland	0.4433 + 0.00006*t	M	1.0528	1.1615	0.9077	0.8779	3.23	0.014	0.0003
Mediterranean shrubland/woodland	0.3953 + 0.00367*t	A	0.0540	0.1093	-0.0127	-0.1506	17.50	0.053	0.0044
Mediterranean forest	0.5551 - 0.00072*t	A	0.0403	0.0277	-0.0468	-0.0211	2.05	0.011	0.0001

A: Additive; M: Multiplicative; t: temporal index of 1 to 28 for the period of 2000 to 2007 in the recurrent seasonal order of Winter (W), Spring (Sp), Summer (Su), and Autumn (A), respectively.

**Table 6.** Decomposition of MODIS EVI time series into trend and seasonal components for natural terrestrial ecosystems in Turkey.

*Natural terrestrial ecosystem types*	*Components*		*Seasonal Indices*				*Accuracy measurements*		
	Trend	Seasonal	W	Sp	Su	A	MAPE	MAD	MSD
(Sub)nival snow/ice	0.0879 + 0.00076*t	A	-0.0799	-0.0810	0.1307	0.0301	22.14	0.017	0.0004
Alpine barren/sparsely vegetated	0.1691 - 0.00016*t	A	-0.0320	-0.0842	0.1130	0.0032	14.35	0.024	0.0015
Boreal barren/sparsely vegetated	0.1781 - 0.001269t	A	0.0004	-0.0535	0.0682	-0.0150	15.03	0.020	0.0007
Boreal forest	0.2234 + 0.00009*t	M	1.0438	0.8409	1.2988	0.8163	6.47	0.014	0.0002
Cool Temperate barren/sparsely vegetated	0.17046 - 0.00026*t	A	-0.0336	0.0364	0.0307	-0.0334	5.57	0.009	0.0001
Cool Temperate grassland	0.1914 - 0.00040*t	M	0.8195	1.1118	1.2276	0.8409	5.32	0.009	0.0001
Cool Temperate shrubland/woodland	0.2108 - 0.00049*t	M	0.8757	1.0178	1.2485	0.8578	4.50	0.008	0.0001
Cool Temperate forest	0.24422 - 0.00040*t	A	-0.0232	-0.0101	0.0621	-0.0288	3.59	0.008	0.0001
Warm Temperate barren/sparsely vegetated	0.1775 + 0.00011*t	A	-0.0347	0.0503	0.0172	-0.0329	4.09	0.007	0.0001
Warm Temperate grassland	0.2267 - 0.00011*t	M	0.9042	1.2371	1.0143	0.8442	3.64	0.008	0.0001
Warm Temperate shrubland/woodland	0.2596 - 0.00036*t	M	0.8574	1.1917	1.1045	0.8462	3.20	0.008	0.0001
Warm Temperate forest	0.2978 - 0.00033*t	M	0.7713	1.0351	1.2301	0.9633	2.44	0.007	0.0001
Mediterranean barren/sparsely vegetated	0.2035 + 0.00017*t	A	-0.0288	0.0773	-0.0161	-0.0324	3.74	0.008	0.0001
Mediterranean grassland	0.2687 + 0.000008*t	A	-0.0174	0.0668	-0.0050	-0.0444	3.93	0.010	0.0001
Mediterranean shrubland/woodland	0.2768 - 0.00003*t	M	0.9280	1.2723	0.9481	0.8514	3.88	0.010	0.0002
Mediterranean forest	0.2935 - 0.00033*t	A	-0.0156	0.0339	-0.0005	-0.0176	2.12	0.006	0.00006

A: Additive; M: Multiplicative; t: temporal index of 1 to 28 for the period of 2000 to 2007 in the recurrent seasonal order of Winter (W), Spring (Sp), Summer (Su), and Autumn (A), respectively.
